# Dynamic Oculomics for Diagnostic Monitoring: Personal Ocular Trends, Risk Stratification and Treatment Response

**DOI:** 10.3390/diagnostics16172699

**Published:** 2026-08-24

**Authors:** Alessandro Avitabile, Mario D. Toro, Roberta Amato, Dario Rusciano, Caterina Gagliano

**Affiliations:** 1 Biomedicine, Neuroscience and Advance Diagnostics (BIND) Department, University of Palermo, 90128 Palermo, Italy; alessandro.avitabile@unipa.it; 2 Neurovisual Science Technology, Research Division (NEST-G.R.I.D.O.), 95123 Catania, Italy; robertaamato@nestweb.it; 3 Eye Clinic, Department of Public Health, University of Naples Federico II, 80131 Naples, Italy; mariodamiano.toro@unina.it; 4 Chair and Department of General and Paediatric Ophthalmology, Medical University of Lublin, 20-079 Lublin, Poland; 5 Department of Special Surgery, University of Jordan, Queen Rania St., Amman 11942, Jordan; 6 Department of Medicine and Surgery, Kore University of Enna, 94100 Enna, Italy; caterina.gagliano@unikore.it; 7 Ophthalmology Clinic, Mediterranean Foundation G.B. Morgagni, 95125 Catania, Italy

**Keywords:** diagnostic monitoring, dynamic oculomics, oculomic kinetics, longitudinal biomarkers, retinal imaging, optical coherence tomography, OCT angiography, continuous intraocular pressure monitoring, aqueous humour, health economics, risk stratification, treatment response

## Abstract

Oculomics uses ocular images, ocular biofluids and functional ocular tests to extract diagnostic information on local or systemic biological states. Most current evidence is cross-sectional: one retinal photograph, OCT scan, OCTA examination or tear sample is compared with a reference population, or converted into a diagnostic or prognostic score. This approach is useful for screening and risk enrichment, but it does not show whether the same patient is changing over time. In this review, dynamic oculomics, or oculomic kinetics, is presented as a diagnostic-monitoring approach based on repeated ocular measurements within the same individual. Its object is the personal trajectory: baseline level, short-term variability, long-term drift, response to stimulus, recovery and modification by treatment. We discuss the biological basis of ocular trajectories, the limits imposed by local ocular regulation and disease, and the technical constraints of fundus photography, structural OCT, OCTA, functional challenge tests, tear analysis, aqueous humour analysis, continuous intraocular pressure monitoring and home-based imaging. We also examine artificial intelligence, validation, governance, health-economic considerations and practical implementation. The available evidence does not support a universal ocular surrogate for systemic disease. More realistic applications include detection of unexpectedly rapid tissue change, individualised risk stratification, treatment-response assessment and recognition of discordance between systemic markers and ocular tissue behaviour.

## 1. Introduction: Why a Single Diagnostic Snapshot Is Not Enough

Oculomics refers to the extraction and interpretation of information from ocular images, ocular biofluids and ocular functional tests in order to infer local or systemic biological states. It has generally started from one examination. A fundus photograph is taken, an optical coherence tomography (OCT) scan is segmented, or an ocular-fluid sample is collected. The resulting measurement is then compared with a population, or related to age, blood pressure, glycaemic status, neurological disease, renal function, or later clinical events. In this way, the eye is used as an accessible diagnostic window. This strategy has undoubtedly been useful. Deep-learning analysis of fundus photographs, for example, has shown that image features not routinely quantified by clinicians contain information about age, sex, smoking, systolic blood pressure and glycated haemoglobin [1].

The first limitation is temporal. A model trained on one retinal photograph can estimate a systemic characteristic or assign future risk, but it cannot tell whether that phenotype has been present for years, has appeared recently, or is continuing to deteriorate. The same limitation persists when the clinical outcome is followed prospectively. Retinal age gap derived from baseline fundus photographs has been associated with subsequent mortality [2], and a deep-learning retinal ageing biomarker has predicted cognitive decline and incident dementia [3]. In both cases, the person was followed over time; the ocular marker was not necessarily followed as a changing biological variable.

This distinction is not a matter of terminology. It is the central point of the present review. A comparison with a normal group tells us whether a value is common or uncommon in a population. A personal trend tells us whether the same value has changed in that individual. These two forms of information can lead to different conclusions. A patient may still fall inside the normal range, but may have lost a relevant amount of retinal tissue compared with his or her previous state. Another patient may remain outside the reference range, but without any evidence of active deterioration.

Population prediction, moreover, is not the same as individual monitoring. In people with diabetes, a baseline retinal vascular profile improved the prediction of incident hypertension, but added little for cardiovascular disease, diabetic kidney disease, or hyperlipidaemia [4]. This example is important because it reminds us that a statistically valid association does not automatically become a clinically useful monitoring tool. Repeating a weak or non-specific measurement may only repeat the same uncertainty at several visits.

There are, nevertheless, settings in which serial ocular measurements have captured progression in a biologically meaningful way. In diabetes, longitudinal imaging has shown neural loss before the vascular lesions traditionally used to define diabetic retinopathy [5]. In multiple sclerosis, the rate of ganglion cell-inner plexiform layer atrophy over almost four years was associated with the rate of whole-brain and grey-matter atrophy [6]. In these examples, the relevant information was not retinal thickness alone, but its change over time.

These observations do not yet justify a general claim that the eye can monitor systemic disease in any context. The diabetic retina is itself a target organ, and retinal thinning in multiple sclerosis is influenced by optic neuritis, disease phenotype, and local axonal injury. Indeed, in the latter study, the relationship between retinal and brain atrophy changed when eyes or patients affected by optic neuritis were excluded [6]. A longitudinal ocular marker may therefore be important without being a clean surrogate for pathology in another organ.

An even earlier difficulty concerns measurement. Retinal fractal dimension has shown different repeatability according to the analytical pipeline applied to the same type of fundus images [7]. In OCT angiography (OCTA), visibility of fine vessels, motion artefacts, image quality, and signal strength influence whether vessel-density measurements are reproducible [8,9]. When the expected biological signal is small, a modest change in acquisition quality may be sufficient to mimic progression.

Nor should every reproducible fluctuation be considered pathological. In healthy eyes, optic nerve head vessel density measured by OCTA differed slightly between morning and evening, whereas macular vessel density was more stable [10]. Signal strength and time of acquisition can therefore affect serial measurements [9,10]. Other physiological influences have to be controlled according to the tissue and modality under investigation, and not assumed to operate in the same way in all measurements.

In this review we therefore address four questions, which are diagnostic before being theoretical. Which ocular variables are sufficiently repeatable for individual longitudinal use? Which changes reflect systemic physiology, and which are more likely to reflect local ocular disease or measurement instability? Does the ocular course add diagnostic information beyond conventional clinical markers? Finally, if an abnormal course is detected, what diagnostic or monitoring action should follow? Static associations are used here only when they help to clarify these dynamic questions.

The purpose is not to assume that the eye is already a reliable longitudinal sensor of systemic health. The purpose is to define, step by step, the conditions under which such a diagnostic claim would become scientifically defensible and clinically useful. In other words, the review moves from a simple observation--the eye can be examined repeatedly--to a more difficult question: when does repeated ocular information become useful diagnostic information for the individual patient?

**Figure 1:**
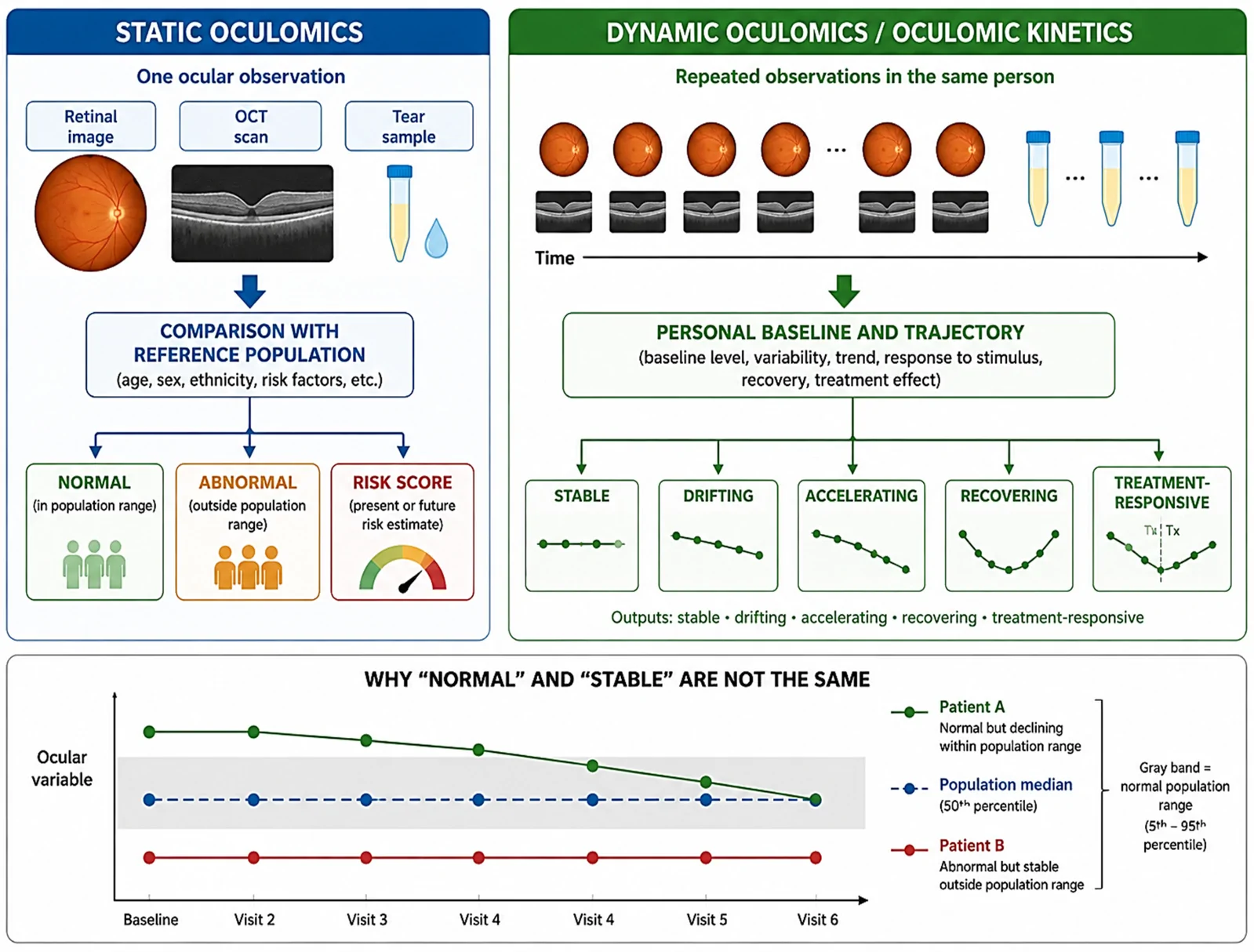
Static versus dynamic oculomics. A single ocular observation supports population-based classification or risk scoring. Repeated observations in the same person define a personal trajectory. The graph shows that “normal” is not necessarily “stable”. The grey shaded band represents the normal population reference range (5th-95th percentile).

Oculomic kinetics, or dynamic oculomics, is used here for the study of how the same ocular variable changes within the same person over time (Figure 1). Its object is not only the value itself, but its baseline, variability, rate of change, response to a stimulus, recovery and modification by treatment. Static oculomics asks whether a measurement is unusual compared with others; dynamic oculomics asks whether the measurement has changed meaningfully for that person.

This distinction places dynamic oculomics within the broader logic of diagnostic medicine. A diagnostic approach does not rely only on whether a patient differs from a reference population, but also on whether that patient is moving away from his or her own previous biological condition. In this sense, oculomic kinetics may provide a practical model of patient-centred diagnostic monitoring, because the eye can be examined repeatedly, non-invasively, and with increasing structural, vascular, functional and molecular resolution.

In this review, dynamic oculomics and oculomic kinetics are used interchangeably to describe the longitudinal analytical approach, whereas ocular temporal phenotype refers to the observed within-person pattern generated by repeated measurements. The principal differences between static and dynamic oculomics are summarized in Table 1.

**Table 1:** Static oculomics and dynamic oculomics: advantages and limitations.

Feature	Static Oculomics	Dynamic Oculomics/Oculomic Kinetics
Main question	Is this value unusual or predictive?	Has this value changed meaningfully in this person?
Comparator	Population reference or risk model	Personal baseline plus expected biological and technical variation
Typical input	One image, scan, sample, or functional test	Repeated images, scans, samples, or functional tests
Main strengths	Scalable; suitable for screening; can use existing archives	Detects individual drift, response, recovery, and discordance
Main limitations	Cannot distinguish old damage from active change	Requires repeatability, standardisation, follow-up, and modelling
Best use	Baseline classification, screening, risk enrichment	Monitoring, treatment response, trial enrichment, personal trend analysis
Main danger	Interpreting prediction as progression	Interpreting measurement noise as biological change

### Literature Search Strategy

The literature for this narrative review was identified primarily through PubMed/MEDLINE, supplemented by backward reference screening and targeted searches of journal and publisher databases. The final updating search was conducted on 18 August 2026. The main Boolean structure combined ocular terms (oculomics OR retinal imaging OR fundus photography OR optical coherence tomography OR OCT OR OCT angiography OR OCTA OR tear biomarkers OR aqueous humour OR ocular fluidomics OR intraocular pressure monitoring) with temporal terms (longitudinal OR serial OR progression OR repeatability OR biological variability OR challenge response OR recovery OR treatment response) and disease or implementation terms (systemic disease OR cardiometabolic disease OR neurodegeneration OR glaucoma OR validation OR artificial intelligence OR health economics). Priority was given to longitudinal human studies, prospective cohorts, intervention studies, and methodological investigations addressing repeatability or temporal variability. Cross-sectional studies were retained when they provided essential biological or technical context. English-language articles indexed up to August 2026 were considered. Initial article selection and reference verification were performed by one author and then checked during manuscript revision by the author group. Each journal article was checked against PubMed, Crossref or the publisher record for authorship, title, bibliographic details, DOI, and correspondence with the statement it supports. Regulatory, legal, and guidance documents were verified against the relevant official institutional source. Study selection followed the conceptual questions of the review rather than a formal systematic-review or meta-analytic protocol. During the preparation of this manuscript, OpenAI ChatGPT (GPT-5.5 Thinking) was used for language editing, formatting, and stylistic revision. Tables and figures were also designed with the help of ChatGPT. The authors reviewed and edited the output and take full responsibility for the content of the published article.

## 2. From a Single Value to the Ocular Temporal Phenotype

Repeated measurements do not all describe the same biological event. A slow reduction in retinal layer thickness, a morning-to-evening fluctuation in choroidal thickness, and a transient vascular response to flickering light are all changes, but they are not the same kind of change. They differ in timescale, mechanism, error structure, and possible clinical meaning. For this reason, it would be misleading to call all of them simply dynamic biomarkers.

We use the expression ocular temporal phenotype as a practical working term for the pattern formed by an ocular variable across time. The term is not meant to introduce a new assay. It is meant to remind us that the biological meaning of a measurement depends not only on its level, but also on its variability, drift, response to perturbation, recovery, and modification by treatment. This is the same logical shift that occurs when one moves from a single blood pressure value to the history of blood pressure control in a patient.

### 2.1. Baseline Level and the Limits of Population Reference Ranges

Clinical measurements are usually interpreted against a reference population. This is necessary because a clinician must first know whether a value is unusually high or unusually low compared with people of similar age or other relevant characteristics. However, a population range is not a personal history. It says where the patient is placed among others, but it does not tell how the patient reached that place.

Laboratory medicine addresses this distinction through the reference change value, which estimates whether the difference between serial results exceeds that expected from analytical imprecision and within-person biological variation [11]. A result can remain inside the population interval yet change substantially for the individual. The reverse is also possible: an unusual value can remain stable without indicating active deterioration.

Ocular measurements add further sources of variance. Scan placement, image quality, magnification, segmentation, media opacity, pupil size, axial length, and the geometry of the tissue being measured can all contribute [7,8,9]. A fixed threshold for meaningful change is therefore unlikely to perform equally well across devices, anatomical regions, and baseline values.

An individual baseline is useful only when its uncertainty is known. One initial scan should not automatically be treated as the true personal reference state. Poor fixation, artefact, transient changes in ocular physiology, or segmentation error may distort all later comparisons. In an ideal situation, the personal baseline should be built from more than one technically adequate measurement. In practice this may not always be possible, but the principle remains important: a trend can be interpreted only if the starting point is credible.

A personalised baseline also does not demonstrate health. A subject examined after years of hypertension, diabetes, or subclinical neurodegeneration may already have an altered retina at the first visit. Population and personal references therefore do different things and should be used together. The population reference describes how unusual the starting level is; the personal reference describes whether that level is moving.

### 2.2. Long-Term Drift: Rate, Direction, and Deviation from Expectation

The simplest descriptor after baseline is the slope. It uses several observations and is less vulnerable than a two-visit difference to one anomalous measurement. Yet a slope remains an estimate, and its interpretation depends on the change expected in healthy tissue.

OCT measurements have shown that age and ocular magnification influence circumpapillary retinal nerve fibre layer thickness and its normative interpretation [12]. A negative slope cannot therefore be labelled pathological merely because it differs from zero. Wu and colleagues further showed that several definitions of OCT progression produced poor specificity when normal ageing was not adequately accommodated [13]. The same distinction has been tested clinically in glaucoma, where trend-based analysis of OCT change detected progression differently from a fixed event-based rule using a 5-micrometre threshold [14]. Although these studies concern glaucoma, the methodological problem applies directly to systemic oculomics.

A single annualised rate can also conceal non-linearity. Stability may be interrupted by an acute vascular or inflammatory event, followed by a new plateau. Averaging across the entire interval would distribute the abrupt loss over years in which no change occurred. Conversely, an apparently rapid early decline may weaken as additional observations are collected.

At minimum, longitudinal analysis should distinguish continuous drift, acceleration or deceleration, and a discrete change point. None should be inferred confidently from two measurements. Closely spaced visits characterise short-term variability but may be uninformative for slow structural decline; widely separated visits extend follow-up while leaving abrupt changes poorly localised. Sampling should follow the expected biology rather than clinic convenience alone.

### 2.3. Short-Term Variability: Noise, Physiology, or Instability?

Short-term variability is usually treated as an obstacle to progression analysis. Random fluctuation widens the confidence interval around a slope and increases the number of observations needed to detect a small persistent change. Yet a regulated tissue is expected to fluctuate, and the organisation of that fluctuation may itself carry information.

The choroid provides a clear warning. In healthy participants, three-dimensional OCT mapping has shown diurnal changes in choroidal and choroidal-sublayer thickness, whereas retinal-layer measurements are comparatively more stable [15]. A morning and afternoon scan could therefore suggest choroidal remodelling when the main explanation is physiological timing rather than disease.

Such findings have two consequences. When long-term drift is the outcome, timing and physiological conditions should be standardised. When regulation itself is the outcome, the same variation should be preserved and measured. Retinal and choroidal studies already show that the importance of time of day differs by tissue and variable [10,15].

Irregular and structured variability cannot be conflated. Irregular variation may arise mainly from acquisition or analysis. Structured variation recurs in relation to time of day, physiological state, or a defined stimulus. Greater visit-to-visit variability cannot be called biological instability until it exceeds technical variance and reproduces under comparable conditions.

### 2.4. Perturbation Response and Recovery

A resting measurement describes the system under one set of conditions. A challenge test asks how it adapts. In metabolic research, this principle is captured by phenotypic flexibility: health may be reflected not only by fasting values but also by the magnitude, coordination, and resolution of the response to a temporary stressor [16]. Applying the same logic to the eye requires challenges that are safe, standardised, and mechanistically interpretable.

Flicker stimulation is an established example. Increased retinal neural activity evokes vascular dilation, and the response curve yields several variables: resting diameter, maximum dilation, time to peak, post-stimulus constriction, and return towards baseline. Their repeatability is not equal. In healthy adults, maximum dilation and constriction calculated from raw data were more reliable than some outputs derived from fixed proprietary time windows, while time-to-peak measures were less stable [17].

Biological interpretation is equally demanding. Pemp and colleagues found reduced flicker-induced retinal vasodilation in type 1 diabetes and in participants with mild hypertension or hypercholesterolaemia. Brachial flow-mediated dilation was also reduced, but its correlation with the retinal arterial response was weak [18]. Within diabetic retinopathy, arteriolar and venular responses decline with increasing disease severity [19]. Reduced dilation may therefore reflect systemic endothelial dysfunction, local neurovascular uncoupling, neural impairment, microvascular damage, or a combination of these processes.

Recovery deserves separate analysis from the peak response. Similar maximum dilation can be followed by different rates or completeness of return to baseline. Whether such recovery kinetics provide a stable measure of regulatory reserve remains largely untested; they cannot be labelled resilience until their repeatability and relationship to outcomes have been established.

### 2.5. Intervention Responsiveness

A temporal biomarker may change after treatment because the disease mechanism has been modified, a pharmacological target has been engaged, systemic physiology has shifted, an ocular adverse effect has occurred, or the measurement has simply fluctuated. Modifiability does not identify the explanation.

Before-and-after comparisons are particularly vulnerable to regression to the mean. Patients are often selected because an initial value is unusually high, low, or rapidly changing; on repetition, an extreme result tends to move towards the average even without effective treatment [20]. Several pretreatment measurements and an appropriate control group may therefore be needed to estimate the trajectory that would otherwise have occurred.

A treatment-responsive ocular marker may support target engagement or biological response, but it should not be labelled a surrogate endpoint unless treatment-induced change in that marker reliably predicts treatment-induced change in a patient-relevant clinical outcome [21].

Four provisional roles are useful and are summarised in Table 2. A state marker varies with current physiological conditions. A strain marker suggests that compensatory mechanisms are being recruited or are losing efficiency. A damage marker records persistent structural or functional loss. A response marker changes after intervention. These are contextual roles rather than mutually exclusive assay classes; the same measurement can occupy different categories over different timescales.

### 2.6. A Provisional Temporal Model

The ocular temporal phenotype can be reduced to seven questions:

Where does the value lie relative to both the individual’s previous measurements and an appropriate population?

How much does it fluctuate under standardised conditions, and is that fluctuation random or structured?

Is there persistent drift beyond expected ageing and measurement error?

Has the trajectory changed abruptly?

How does the ocular system respond to a defined physiological or therapeutic perturbation?

Does it return towards baseline, and over what interval?

Does the ocular trajectory precede, parallel, or remain discordant with systemic disease activity and patient-relevant outcomes?

The final question sets the clinical threshold. A technically reliable trajectory may describe local ocular physiology well yet add little to systemic assessment. Conversely, a modest signal may matter if it consistently identifies tissue-level change that established markers miss.

**Figure 2:**
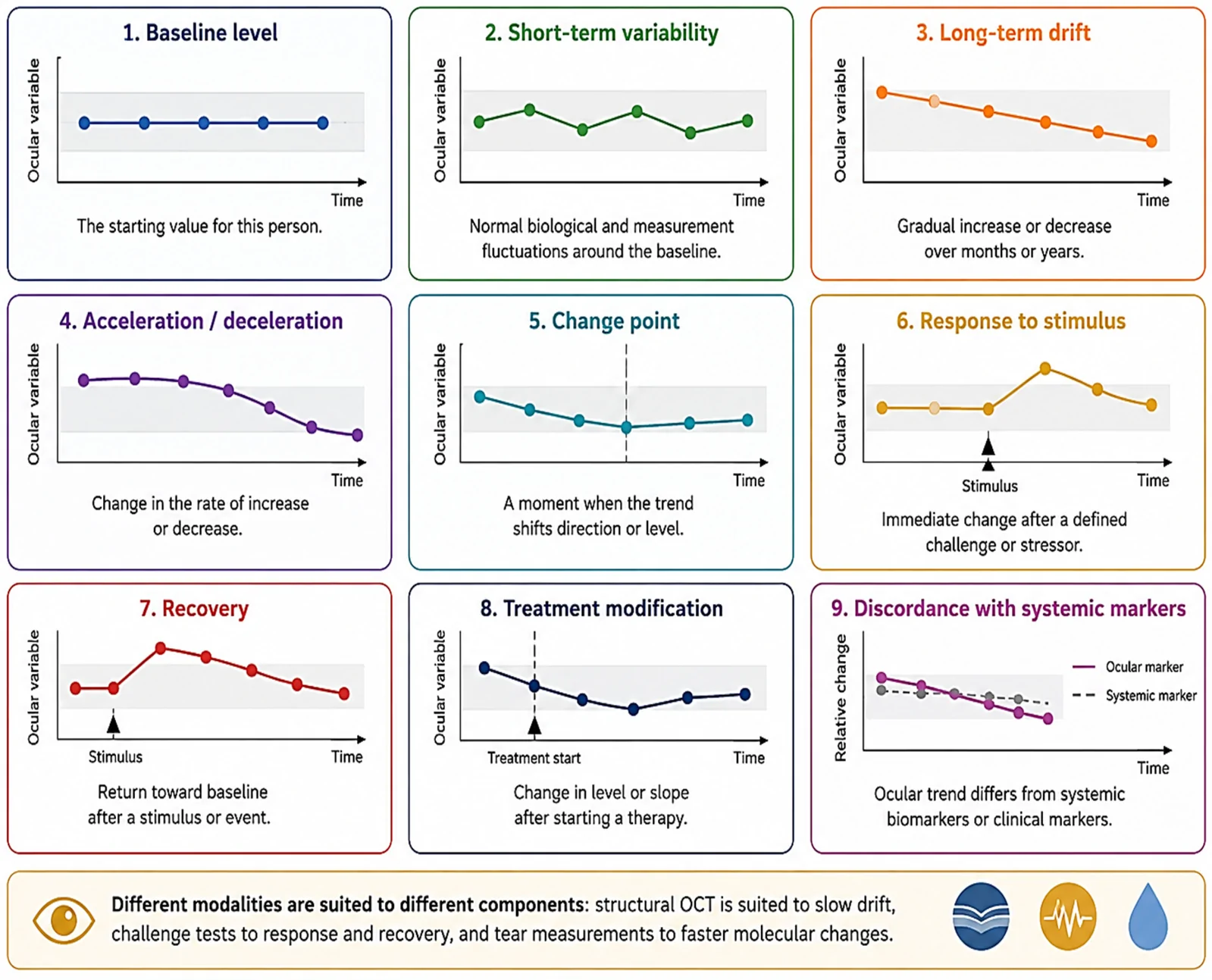
Components of the ocular temporal phenotype. Repeated measurements define baseline, variability, drift, acceleration, change point, stimulus response, recovery, treatment modification and discordance. Bottom symbols indicate imaging/OCT-OCTA, functional testing and tear-biomarker modalities. Grey shaded bandsschematically indicate the expected/reference range of normalbiological and measurement variability and do not represent a fixedquantitative threshold.

The practical consequence is straightforward. When the question is diagnosis, a population comparison may be sufficient. When the question is monitoring, the patient becomes his or her own comparator. Dynamic oculomics is built on this second logic (Figure 2).

## 3. Biological Basis of Ocular Dynamics: Regulation, Compensation, and Injury

The retina is often described as a sensor of systemic health. The analogy is useful, but it must be handled with caution. A sensor, in the strict sense, should respond to an input in a predictable way. Ocular tissues do not behave so simply. They receive several systemic inputs at the same time and transform them through local regulation. Blood pressure, blood gases, glucose availability, autonomic activity, inflammation, neural stimulation, and treatment exposure may all influence the measurement obtained at a particular visit.

A stable retinal value may therefore indicate stable systemic health, but it may also indicate successful compensation despite increasing stress. Conversely, an altered vascular response may precede structural injury, accompany it, or arise from a local ocular disorder. The biological problem is to separate these possibilities. Without this separation, dynamic oculomics would only replace one oversimplification—the single snapshot—with another, namely the uncritical interpretation of every change as systemic disease.

### 3.1. The Retina as a Regulated Neurovascular Tissue

Retinal activity is inseparable from retinal metabolism. The inner retina is supplied predominantly by the retinal circulation, whereas the outer retina depends largely on the choroid. These vascular beds have different haemodynamic and regulatory properties, and methods used to assess retinal or choroidal flow do not measure identical biological processes [22].

A change in a superficial retinal OCTA metric cannot therefore be assumed to have the same meaning as a change in choroidal thickness or perfusion. Even within the retinal circulation, capillary perfusion is spatially and temporally heterogeneous [23]. A global vessel-density value compresses this regional behaviour into one number.

Flickering light increases retinal metabolic demand and normally induces functional hyperaemia. Neurons, Müller cells, astrocytes, pericytes, endothelial cells, and several vasoactive mediators have been implicated, but their relative roles remain context-dependent and can be altered in diabetic retinal disease [24]. Glial signalling, vascular smooth muscle, endothelial mediators and capillary regulation may all contribute. An attenuated response may therefore demonstrate dysfunction without locating it precisely.

The neurovascular unit is not anatomically uniform. Müller-cell associations with vessels differ among the superficial, intermediate, and deep vascular plexuses, and opportunities for direct neural–vascular interaction vary by layer [25]. Experimental imaging has also identified interpericyte tunnelling nanotubes that coordinate light-evoked changes in capillary flow; their disruption after ischaemic injury impairs this communication [26]. These findings make layer-specific and network-level responses biologically plausible, even though they remain difficult to resolve in human clinical imaging.

### 3.2. Compensation May Conceal Disease Before It Reveals It

Retinal autoregulation buffers moderate changes in perfusion pressure, while adjustments in vessel tone, oxygen extraction, and local metabolism support neural function [22,27]. Stability under these conditions is active rather than passive. Resting measurements may remain unchanged while the reserve needed to maintain them is declining.

Challenge tests are attractive because they may expose this loss of reserve, but the resulting signal is not organ-independent. As noted above, the weak correspondence between retinal flicker responses and brachial flow-mediated dilation [18] argues against treating the retinal response as a direct surrogate for peripheral endothelial function. Its potential value may lie in the additional neural, glial, and local microvascular processes incorporated into retinal neurovascular coupling.

Compensation also changes with disease stage. Early shifts in flow, oxygen extraction, or glial support may preserve tissue function. Later, the same system may show reduced reactivity, barrier leakage, capillary loss, and neural thinning. Diabetic retinal disease illustrates the overlap: endothelial dysfunction, pericyte loss, neuronal stress, Müller-cell activation, microglial responses, barrier disruption, and metabolic injury interact rather than follow a single invariant sequence [28]. A marker sensitive to one component may not track the entire course.

### 3.3. Barrier Regulation and Metabolic Exchange Generate Distinct Temporal Signals

The inner blood–retinal barrier depends on endothelial junctions, restricted transcellular transport, and signalling from pericytes, glial cells, and neighbouring neural tissue [29]. It is actively regulated, and early dysfunction can precede visible fluid accumulation.

Structural thickening is therefore a delayed and non-specific consequence of disturbed fluid balance. Conversely, reduced thickness after treatment does not demonstrate that barrier biology has normalised; fluid may resolve while inflammatory or metabolic stress persists [28,29,30].

Glial responses further complicate the sequence. Müller cells regulate extracellular ions, neurotransmitter recycling, water movement, energy exchange, and vascular signalling. Astrocytes support vessels and axons, while microglia can move from surveillance towards inflammatory and phagocytic states. These reactions may initially protect tissue but contribute to dysfunction when prolonged [30].

Metabolism varies by layer and visual state. Photoreceptor oxygen consumption is greatest in darkness, whereas flickering stimulation increases inner retinal demand [27]. Capillary flow is also intrinsically heterogeneous because of regional demand, intermittent erythrocyte passage, and local vascular control [23]. Technical reproducibility alone cannot remove these biological sources of variation.

### 3.4. Reversible Dysfunction and Structural Loss Are Not Successive Forms of the Same Marker

A convenient model places functional disturbance early and structural alteration late, with one progressing smoothly into the other. That sequence may occur, but it is not universal and need not produce a single coherent trajectory.

Vascular reactivity can change rapidly with blood pressure, glycaemia, oxygenation, autonomic activity, or medication. Neural tissue lost through axonal degeneration will not recover on the same timescale, even if perfusion improves. Stable layer thickness over a short interval does not exclude continuing mitochondrial stress, synaptic dysfunction, barrier impairment, or inflammation [27,28,30].

Different measurements may therefore move in opposite directions without either being erroneous. A treatment may normalise vascular reactivity while structural loss continues because earlier injury has become irreversible. Retinal thickness may stabilise while inflammatory or metabolic signals remain abnormal. Recovery should refer to the variable measured: recovery of vessel calibre is not recovery of the retina, and resolution of oedema is not restoration of neural integrity.

### 3.5. Ocular Dynamics Are Shaped by Local Disease

Every ocular signal originates in tissue with its own diseases, exposures, and treatment history. Diabetic retinal disease, previous optic neuritis, age-related neural loss, and physiological choroidal variation can all alter structural or vascular trajectories [5,6,12,15,28,29,30]. A new local event may create an abrupt change that could otherwise be mistaken for systemic deterioration.

Age is similarly difficult to classify. Neural loss, vascular stiffening, mitochondrial impairment, glial change, and choroidal remodelling may parallel systemic ageing without occurring at the same rate. An ocular measurement associated with chronological age does not, by itself, quantify whole-body biological ageing.

Systemic biomarkers may legitimately reflect or interact with local ocular processes. The key criterion is that local influences can be accounted for—whether through measurement, exclusion, stratification, or incorporation—without losing the signal of interest. This standard tightens considerably when interpreting an individual’s longitudinal trajectory rather than a cross-sectional group difference.

### 3.6. Persistence and Biological Memory

Some retinal changes persist after the initiating exposure has improved. In the DCCT/EDIC cohort, participants originally assigned to intensive glycaemic treatment retained a lower cumulative risk of retinopathy progression for years after glycated haemoglobin levels in the former treatment groups had converged [31]. Current glycaemia alone did not explain subsequent retinal outcomes.

This metabolic memory has been linked to persistent mitochondrial injury, oxidative stress, advanced glycation, inflammatory signalling, and epigenetic modifications, although no single mechanism explains the clinical effect fully [32].

Persistence cuts in two directions for dynamic oculomics. Structural loss or enduring lesions may record past systemic injury no longer evident in current laboratory measurements. The same persistence can make the ocular phenotype slow to improve after systemic control, creating apparent discordance between present physiology and tissue outcome. Whether subtler oculomic features retain similarly interpretable memories of hypertension, renal dysfunction, or neurodegeneration remains unproven.

### 3.7. Conditions for Mechanistic Interpretation

A dynamic ocular marker becomes mechanistically persuasive when it is linked to a defined tissue compartment or physiological process; when its main physiological determinants are known; when local ocular causes of change are addressed; and when the sampling interval matches the process being measured. Vascular tone may change within minutes, inflammatory and molecular signals over hours or days, and capillary loss or neural thinning over much longer periods.

The ocular trajectory should also be compared directly with the systemic process it is claimed to represent. Parallel change supports correspondence but does not demonstrate surrogacy. An earlier ocular signal is useful only if it predicts a relevant transition; persistent discordance is informative only after measurement instability and local ocular disease have been excluded.

Ocular tissues are responsive to systemic disturbance but retain enough local autonomy to reshape that disturbance. Their value as longitudinal markers depends on acknowledging both properties.

## 4. Measuring Ocular Change: What Each Modality Can and Cannot Resolve

Repeated acquisition does not automatically convert a measurement into a longitudinal biomarker. The reasoning must begin from the measurement itself. If the expected biological change is smaller than the error of the instrument, the trajectory cannot be interpreted at the individual level. If the measurement is influenced by time of day, image quality, or software version, these factors must be known before the observed difference is called progression. For this reason, a simpler variable with well-defined error may be more useful than a sophisticated feature whose variability has not been characterised.

### 4.1. Fundus Photography: Scalable, Stable, but Not Metrically Neutral

Colour fundus photography is widely available, relatively inexpensive, and compatible with retrospective analysis because screening programmes and epidemiological cohorts already contain large image archives. Vessel calibre, tortuosity, branching geometry, fractal dimension, lesion appearance, and optic-disc characteristics can all be assessed repeatedly.

A vessel measured in pixels has no fixed physical width unless image scale is known. Camera optics, refractive error, axial length, field of view, resolution, and image centration can alter quantitative vascular measurements [33]. Analytical software may be highly repeatable when applied twice to the same image while remaining biassed across images acquired under different conditions.

Such a distinction was evident in comparisons of retinal fractal-dimension pipelines: repeatability and robustness were not uniform across methods and image sets [7]. Unless the analytical pipeline is fixed and documented, part of the observed trajectory may belong to the software rather than the patient.

Long-term archives introduce additional discontinuities. Film may be digitised, cameras replaced, and disc-centred images followed by macula-centred images. In a long-term retinopathy dataset, vessel estimates changed with image-conversion factor and centration, and some geometric variables were more vulnerable than others [33]. Registration can reduce regional mismatch but cannot recreate resolution or vessel visibility absent from the original image.

Fundus photography is best suited to persistent vascular geometry and lesion development rather than rapid physiological responses. Its scale makes it attractive for opportunistic monitoring, but only when acquisition metadata and preprocessing are retained.

### 4.2. Structural OCT: Precision Does Not Eliminate Longitudinal Artefact

Structural OCT is the most mature platform for measuring gradual ocular change. Retinal nerve fibre layer thickness, ganglion cell-inner plexiform layer thickness, total retinal thickness, photoreceptor integrity, choroidal thickness, and focal lesions can be evaluated at micrometre resolution. Longitudinal studies have already quantified age-related retinal thinning [12].

OCT devices nevertheless differ in wavelength, scan speed, geometry, axial resolution, anatomical definitions, segmentation algorithms, and normative databases [34]. Systems reporting a variable under the same name may not delineate identical boundaries. Hou and colleagues found high repeatability and generally strong agreement between swept-source and spectral-domain devices from the same manufacturer, but such results cannot be generalised automatically to other platforms [34].

In practical terms, the safest design retains the same device, protocol, and software version. When equipment has to be changed, overlapping examinations on both systems are preferable to a conversion equation derived only at group level.

Segmentation errors deserve particular scrutiny. Media opacity, low signal, high myopia, epiretinal membranes, oedema, drusen, vascular shadowing, optic-disc anomalies, and advanced tissue loss can all displace automated boundaries [35]. Nagarkatti-Gude and colleagues showed that retinal nerve fibre layer errors may persist over time, producing a consistent but anatomically incorrect trajectory [35]. Reviewing only numerical outputs or progression graphs can therefore miss a repeated error visible on the B-scans.

Manual correction introduces reader dependence and may not be reproducible when images are reanalysed years later. Longitudinal studies should state whether segmentation was accepted automatically, corrected manually, or processed externally. Corrections should be performed without knowledge of systemic outcome whenever feasible.

Scan registration improves precision but depends on stable fixation and recognisable landmarks. Broad averages are generally more repeatable than narrow sectors, although they dilute focal change. Choroidal measurement adds uncertain posterior boundaries and substantial diurnal variation [15]. A difference in several tens of micrometres may reflect acquisition time or haemodynamic state rather than remodelling.

Structural OCT is therefore well suited to persistent drift and cumulative injury. Its numerical precision becomes misleading when anatomical validity and protocol continuity are assumed rather than checked.

### 4.3. OCT Angiography: Vascular Information with a Higher Technical Burden

OCTA visualises retinal and choroidal microvascular networks without injected contrast. Vessel density, perfusion density, foveal avascular zone dimensions, non-perfusion, flow voids, and plexus-specific geometry can be quantified. Conventional clinical OCTA, however, detects motion contrast rather than measuring blood flow directly.

Blood cells must move sufficiently between repeated scans to be detected, while movement of the eye itself creates artefact. Very slow flow may fall below the detection threshold and appear absent; faster flow does not generate a proportional signal. Apparent non-perfusion can therefore reflect vascular biology, slow flow, segmentation, or image quality [8,9,36].

Repeatability varies among metrics. Fine-vessel visibility and image quality influence capillary-plexus density measurements [8], while long-term measurements in healthy eyes are weakened by differences in signal strength [9]. In stable glaucoma and glaucoma-suspect cohorts, macular and optic-nerve-head vascular parameters were reproducible, but generally more variable than structural OCT thickness measures [36].

Software is particularly consequential. Projection-artefact removal, thresholding, skeletonisation, scan size, inclusion of large vessels, and segmentation boundaries can change without altering the name of the output variable. Raw data and software versions should be retained where possible. Plexus-specific analysis is biologically appealing but technically vulnerable because structural segmentation errors propagate directly into the en face vascular slab.

OCTA can track microvascular drift and short-term responses, but it cannot be described as a direct map of capillary perfusion without qualification.

### 4.4. Functional and Challenge-Based Measurements

Structural imaging asks what tissue is present. Functional testing asks what the ocular system can do at rest or under challenge. Pupillary responses, contrast sensitivity, colour discrimination, electrophysiology, vascular reactivity, and dark adaptation operate over different timescales and cannot be reduced to one generic functional outcome.

Dynamic vessel analysis illustrates the problem. Maximum dilation, time to peak, post-flicker constriction, baseline fluctuation, and recovery describe different parts of the response. Their repeatability varies, and some variables calculated from raw response curves outperform values generated through fixed analysis windows [17].

The stimulus should also be controlled. Flicker frequency, luminance, duration, adaptation, pupil size, fixation, and baseline vessel diameter influence the curve. Systemic physiological state and vasoactive exposures can add further variation [18,22,27]. Challenge testing interrogates reserve rather than resting level, but the response remains a mixture of neural activation, glial signalling, vascular behaviour, endothelial function, and autonomic state.

### 4.5. Tear-Fluid Analysis: Repeated Access, Unstable Composition

Tears can be collected repeatedly with little burden and contain proteins, cytokines, and other molecular constituents that may respond to exposure, inflammation, or treatment. The attraction of rapid molecular readouts is offset by the fact that the sample is altered by the way it is collected.

Capillary tubes mainly collect free tear fluid, whereas Schirmer strips contact the conjunctival surface and recover surface-associated and intracellular proteins in addition to soluble components. Paired sampling has produced systematic differences in proteomic profiles [37]. Collection duration, reflex tearing, strip position, topical anaesthetic, storage, freeze–thaw cycles, extraction buffer, and assay platform can all alter the result [37,38].

Temporal stability is analyte-specific. Some tear cytokines are comparatively stable, while others show intra-day or inter-day variation [38]. Normalisation is also unsettled: concentration per unit volume is difficult when volume is uncertain, total-protein normalisation may obscure genuine changes in overall abundance, and Schirmer wetting length is only an indirect estimate of dilution.

Repeated tear analysis may be best suited to changes over hours, days, or weeks. Its longitudinal maturity remains lower than that of structural imaging because many proposed biomarkers come from cross-sectional comparisons and lack test–retest data.

### 4.6. Aqueous Humour Analysis: Molecular Information with Limited Longitudinal Accessibility

Aqueous humour is another important ocular biofluid for molecular profiling, especially in anterior-segment disease, glaucoma, uveitis and retinal conditions treated surgically or by intravitreal procedures. Compared with tears, it is anatomically closer to intraocular tissues and may better reflect chamber-specific inflammatory, metabolic, proteomic, elemental or transcriptomic processes. Recent aqueous and vitreous humour proteomic studies in uveitis, multi-elemental aqueous-humour analyses in patients with cataract and cardiometabolic comorbidities, and multi-omics glaucoma studies combining transcriptomic or chromatin-accessibility analyses with aqueous-humour validation illustrate the potential of this compartment for diagnostic molecular profiling [39,40,41].

Its place in dynamic oculomics is nevertheless narrower than that of imaging or tear sampling. Aqueous humour collection is invasive, ethically justifiable mainly when linked to clinically indicated surgery or intraocular treatment, and usually unsuitable for dense repeated monitoring in otherwise stable patients. Longitudinal aqueous-humour studies may therefore be most informative in selected contexts, such as treatment-response studies, disease-stage comparisons, or opportunistic sampling during repeated intraocular procedures, rather than as routine surveillance. Sampling time, surgical indication, topical or intraocular therapy, blood-aqueous-barrier status, storage and assay platform should be recorded because they can influence the apparent molecular trajectory.

### 4.7. Continuous Intraocular Pressure Monitoring: A True Ocular Time Series with a Narrow Disease Context

Intraocular pressure (IOP) deserves specific mention because it is one of the few ocular variables for which continuous or quasi-continuous monitoring has already been developed. Office-hour tonometry provides isolated measurements and can miss nocturnal peaks or circadian patterns. Contact lens sensor systems do not measure IOP in millimetres of mercury, but they can record a pressure-related signal over 24 h and thereby convert a static measurement into a temporal profile.

Mansouri and colleagues analysed repeated 24 h contact lens sensor recordings in glaucoma and glaucoma-suspect patients and showed that circadian pattern modelling could identify reproducible features, including nocturnal acrophase in a substantial proportion of participants [42]. De Moraes and colleagues later reported that variables derived from 24 h contact lens monitoring were associated with visual-field progression and added information beyond Goldmann IOP measurements obtained during follow-up [43].

Continuous IOP monitoring therefore supports the central argument of dynamic oculomics: a single clinic value may be less informative than the behaviour of the variable across time. Its limits are equally instructive. The signal is device-specific, expressed in arbitrary units, affected by ocular surface and contact-lens tolerance, and mainly validated in glaucoma. It is a strong example of ocular kinetics, but not a general systemic oculomic marker.

### 4.8. Portable and Home-Based Acquisition: Temporal Density Versus Control

Clinic-based measurements offer controlled acquisition but sparse sampling. Portable and home devices provide denser time series while moving acquisition away from trained operators and standardised environments.

Early work with a portable sparse-OCT prototype in an older age-related macular degeneration cohort showed that repeated retinal imaging was feasible and that central retinal thickness measurements were broadly comparable with those obtained using a conventional device [44]. The limits of agreement were nevertheless wide enough to matter when small changes are interpreted.

A later DRCR Retina Network study evaluated daily home OCT in newly diagnosed neovascular age-related macular degeneration. Among participants who initiated scanning, adherence and the proportion of quantifiable scans were high, and daily imaging revealed marked differences in fluid trajectories after treatment [45]. Uptake was more limited, showing that connectivity, willingness, physical ability, and familiarity with the device become part of the data-generating process.

These studies concern local retinal disease rather than systemic oculomics. Their relevance is that dense self-acquired ocular time series are feasible. Whether the same approach can resolve subtler systemic signals is unknown. Daily acquisition also multiplies artefacts, making automated quality control and models that account for autocorrelation and unequal adherence essential.

### 4.9. Selecting a Modality According to the Temporal Question

The modality should be chosen after the biological process and timescale have been specified. Annual structural OCT is poorly matched to a transient inflammatory response; repeated tear or aqueous-humour sampling is unnecessary for estimating slow neuroaxonal loss; and a vascular challenge test cannot substitute for a measure of cumulative tissue damage.

Multimodal acquisition is useful when measurements answer complementary questions, such as whether reduced vascular reactivity precedes structural loss. Adding variables without a temporal or mechanistic rationale merely adds error distributions, missingness, and incompatible sampling requirements.

Before an ocular trajectory is linked to a systemic outcome, four modality-specific quantities should be established: short-term repeatability, expected physiological variability, minimal detectable change, and stability after changes in hardware or software. These four quantities form the technical basis for personal trend analysis. Without them, the longitudinal signal remains inseparable from the way it was measured. The final column of Table 3 uses descriptive evidence categories rather than a formal grading system, because the maturity of evidence differs markedly across modalities and intended uses.

## 5. Diagnostic Use Cases: When Does an Ocular Trajectory Add Information?

Diagnostic relevance begins when change in an ocular variable alters an inference that could not be made from its current value alone. This point deserves emphasis. A retinal feature may be associated with disease, but this does not necessarily mean that its evolution in one patient can guide diagnosis, risk stratification or follow-up. To be useful, the ocular measurement must change reproducibly, the direction of change must be biologically plausible, and the information must improve on what is already known from symptoms, examination, laboratory tests, or imaging of the affected organ.

The available evidence is uneven, and this is not surprising. Serial retinal measurements are well established in ophthalmic disease and have gained a defined role in multiple sclerosis, where the retina itself provides neuroaxonal information. Their use for broader systemic monitoring is less mature. Cardiometabolic cohorts show that retinal vessels change with systemic exposure, but the changes are not disease-specific. Neurodegenerative studies reveal associations between retinal and clinical trajectories, although not always in the expected direction. Intervention studies show that ocular variables can be modified, but they do not by themselves validate these variables as surrogates.

### 5.1. Biological Ageing: A Trajectory Cannot Be Inferred from an Age Score

Retinal age models have helped establish that fundus images contain information associated with mortality and cognitive outcomes [2,3]. These results are relevant to the present discussion, but they should be placed in their proper category. They are mainly evidence that one image can contain an accumulated biological signature. They are not, by themselves, evidence that retinal ageing has been measured as a personal longitudinal process.

Structural OCT studies provide age- and magnification-dependent reference information for retinal nerve fibre layer thickness [12], but applying a mean population behaviour to an individual remains problematic. Ageing effects vary with baseline anatomy, ocular geometry and retinal location, while short series can generate apparently abnormal slopes through measurement error [13]. A retinal-age algorithm trained on cross-sectional data adds another layer of uncertainty: a change in predicted age may reflect genuine tissue evolution, variation in image acquisition, or instability of the model itself.

The clinically useful quantity is unlikely to be ‘retinal age’ alone. A more useful quantity may be departure from an expected personal slope, acceleration after a period of stability, or failure to recover after a defined physiological stress. None of these has yet been qualified as a general marker of biological ageing. At present, retinal-age estimates are best regarded as tools for risk enrichment, not as substitutes for longitudinal observation.

### 5.2. Cardiometabolic Trajectories: Reciprocal Change Without Diagnostic Specificity

The cardiometabolic setting provides the clearest evidence that systemic exposure and retinal vascular phenotype evolve together. Longstanding blood pressure, glycaemic burden, adiposity, smoking, medication, and inflammation all affect the microcirculation. The advantage of serial retinal imaging is that it can examine whether vascular geometry changes as these exposures change, rather than comparing different individuals at one time point.

Recent data from the Canadian Longitudinal Study on Ageing illustrate both the promise and the limitation of this approach. Over approximately three years, blood pressure, smoking, obesity, and diabetes were associated with changes in retinal vessel diameter, area, or tortuosity. Diastolic blood pressure showed longitudinal associations with several arteriolar and venular traits, while type 1 diabetes was associated with arteriolar widening [46]. The study supports biological responsiveness of retinal vessels, but it does not demonstrate a single vascular pattern that identifies one systemic process. Several exposures converged on overlapping measurements.

The relation may also be bidirectional. In a four-year childhood cohort, narrower retinal arterioles at baseline were associated with higher subsequent systolic blood pressure, while higher baseline blood pressure predicted later arteriolar narrowing [47]. This is more informative than a one-directional prediction model because it suggests an evolving interaction between systemic pressure and microvascular structure. It remains unclear whether the retinal alteration contributes to rising blood pressure, reflects the same vascular process, or simply records cumulative haemodynamic exposure.

Diabetes offers stronger biological anchoring because the retina is itself a target organ. Serial neural loss before visible retinopathy [5] and the long-term retinal consequences of earlier glycaemic exposure [31,32] show that ocular trajectories can record both current and historical metabolic injury. That strength is also a limitation for systemic inference. A worsening retinal trajectory may indicate local diabetic retinal disease more directly than whole-body microvascular deterioration.

The most plausible cardiometabolic role is not diagnosis from a retinal slope. It is the detection of unexpected tissue behaviour: deterioration despite apparently satisfactory risk-factor control, unusually rapid change in a person with borderline systemic measurements, or improvement that accompanies a sustained intervention. Those uses require prospective comparison with conventional biomarkers.

### 5.3. Neurodegeneration: Linked Slopes Do Not Necessarily Move in Parallel

Repeated OCT has a more advanced position in neurological disease because the measurement targets neuroaxonal tissue rather than an indirect vascular correlate. In multiple sclerosis, ganglion cell–inner plexiform layer loss has been associated with concurrent brain atrophy [6]. Even there, optic neuritis and local optic-pathway injury affect the relationship. The retina supplies a complementary measure of neurodegeneration; it is not a neutral sample of the brain.

Parkinson’s disease exposes a less intuitive problem. In a prospective cohort, parafoveal ganglion cell-inner plexiform layer thinning was faster in patients than in controls, yet patients with more advanced baseline atrophy showed slower subsequent macular thinning and more rapid cognitive decline [48]. The apparently reassuring slope may have reflected a floor effect or deceleration after substantial loss.

The finding matters because rate cannot be interpreted apart from the starting point and remaining dynamic range. Once a retinal layer is markedly thinned, additional neural injury may be poorly expressed by that variable; a low rate of change can then coexist with worsening clinical disease.

Longitudinal retinal loss has also been reported after remote mild traumatic brain injury. Veterans with a history of mild traumatic brain injury showed greater retinal nerve fibre layer thinning than controls, accompanied by deterioration in visual-field and contrast-sensitivity measures; retinal loss was modestly related to cognitive change and injury severity [49]. The result supports persistent neural change after an apparently discrete event. It does not determine whether the measured loss arose from progressive central neurodegeneration, chronic optic-pathway injury, or both.

These studies favour trajectory analysis over universal cut-offs, but they also show why trajectories cannot be interpreted without disease stage. The real question is not simply whether retinal tissue is thinning. It is whether the observed rate, anatomical distribution, and remaining measurement range correspond to a clinically meaningful phase of the neurological disorder.

### 5.4. Treatment Response: Modifiable Does Not Mean Validated

Intervention studies provide a more direct test of ocular responsiveness because the timing of exposure is defined. They can show that an ocular variable changes after treatment and whether the change differs from that in a control group. What they cannot establish automatically is that the ocular response mediates or predicts clinical benefit.

Two reports from the same randomised EXAMIN AGE programme are instructive. Twelve weeks of high-intensity interval training produced arteriolar widening and venular narrowing, together with changes in oxidative-stress-related measures [50]. A parallel analysis found improved flicker-induced arteriolar dilation but no significant venular response [51]. These are complementary observations from one programme, not independent replications.

Resting calibre and stimulus-evoked reactivity did not behave as interchangeable measures. Some ocular associations persisted after adjustment for changes in body composition, blood pressure, medication, or cardiorespiratory fitness [50,51], although such adjustment does not isolate a separate retinal causal pathway.

A different intervention model comes from bariatric surgery. In a small longitudinal study, retinal vascular measures, peripheral microvascular function, and cardiovascular risk variables were assessed before and one year after Roux-en-Y gastric bypass [52]. Several measures improved after substantial weight loss. The design demonstrates feasibility of aligning ocular and systemic changes, but the absence of a contemporaneous untreated control and the scale of metabolic change limit causal attribution to any single pathway.

A similar caution applies to imaging phenotypes that appear clinically coherent but may represent different disease mechanisms. In age-related macular degeneration, multimodal analysis has distinguished macular atrophy secondary to neovascular disease from de novo geographic atrophy and showed different baseline atrophic area, progression and OCT biomarker profiles [53]. This type of evidence is useful because it separates an imaging phenotype from mechanism, previous treatment exposure and longitudinal clinical outcome.

Treatment monitoring will require more than before-and-after significance. A candidate ocular response marker should show a dose or exposure relationship, reproduce in an independent trial, and predict a subsequent outcome or treatment benefit beyond conventional measures. The expected timing also matters. Vascular reactivity may improve before structural remodelling; neural loss may stabilise without recovering; persistent damage may remain visible despite correction of the initiating systemic disturbance.

### 5.5. Discordance May Be More Useful than Agreement

Close agreement with systemic markers is not always the most informative result. It confirms correspondence but may add little to measurements already available. Discordance raises a harder, and sometimes more useful, question.

Continued retinal deterioration despite improved glycated haemoglobin, controlled blood pressure, or reduced inflammatory markers could indicate residual tissue risk, previous irreversible injury, inadequate duration of control, or an unrecognised local ocular process. Apparent ocular improvement without systemic benefit may reflect a rapidly reversible vascular state rather than modification of cumulative disease. Neither pattern should be labelled treatment failure or success without further investigation.

The immediate diagnostic roles are therefore narrower than universal systemic surveillance. Serial ocular measurements may help enrich clinical trials for participants with demonstrable tissue progression, provide an accessible secondary endpoint, identify unexpected divergence between risk-factor control and tissue behaviour, or prompt diagnostic reassessment when deterioration exceeds measurement error. Surrogate-endpoint status would require evidence that treatment-induced change in the ocular marker predicts treatment-induced change in a patient-relevant outcome [21].

Only selected applications approach this standard. Evidence is strongest where ocular tissue is directly involved in the disease and repeated measurements can be interpreted beside organ-specific clinical data. Using the eye to monitor otherwise unrelated systemic disorders remains a proposition to be tested, not an established service.

## 6. Artificial Intelligence for Longitudinal Oculomics: Forecasting Is Not the Same as Following Change

Artificial intelligence entered oculomics largely through single-image prediction. A retinal photograph could be used to estimate age, blood pressure, smoking status, or future cardiovascular risk [1]. Later models linked a baseline image-derived age estimate with mortality or cognitive decline [2,3]. These studies were important because they showed that the retinal image contains information not easily appreciated by direct inspection. They did not, however, require the algorithm to understand a personal course over time.

This distinction is easy to blur, but it is essential. A model may be trained in a longitudinal cohort and predict an event several years ahead while still receiving only one image from each person. The follow-up data provide the outcome label, not necessarily the temporal input. Such a model forecasts from a snapshot. A dynamic model must do something different: it must interpret a sequence and decide what has remained stable, what has changed, how fast it has changed, and whether the pattern departs from the previous personal course.

### 6.1. From Baseline Risk to an Observed Course

DeepDR Plus illustrates the distinction. It used baseline fundus photographs to estimate time to diabetic retinopathy onset or progression and performed well across several longitudinal cohorts [54]. The model may help tailor screening intervals, yet it does not observe the retinal course that follows the baseline image. A later photograph can generate a new score, but the difference between scores has no longitudinal meaning until test–retest behaviour and sensitivity to acquisition changes have been established.

Serial information can improve forecasting when it is used explicitly. In glaucoma, Herbert and colleagues combined early visual-field, OCT, and clinical observations to predict rapid future worsening; models that incorporated subsequent early visits outperformed those based on baseline alone [55]. Even here, the algorithm may learn more than tissue progression. Visit frequency, treatment intensity, and inconsistency among early tests can all encode clinical concern. A useful model should be compared with the latest value, an ordinary slope, and clinician judgement rather than only with another neural network.

The observation window creates a practical trade-off. More visits improve estimation, but waiting for them postpones the decision. A model that needs years of data to forecast the next year may be statistically elegant and clinically late.

### 6.2. Registration, Data Scarcity, and Temporal Leakage

Before an algorithm interprets change, it should compare the same tissue. Fundus images vary in scale, illumination, field position, and eye rotation; OCT and OCTA series add scan placement, segmentation, and signal-strength effects [8,9,33,34,35,36]. If camera replacement coincides with follow-up time, a model can learn the acquisition era rather than vascular remodelling.

EyeLiner uses retinal landmarks and deep-learning keypoint matching to align serial colour photographs and reduced landmark misalignment across three longitudinal image sets [56]. Registration improves comparison but is not neutral. Excessive deformation may suppress genuine lesion growth, whereas inadequate alignment leaves technical displacement. Original and registered images should both be retained, and the transformation should remain inspectable.

Longitudinal datasets are small because they require repeated imaging, stable patient linkage, retention, treatment documentation, and enough events to separate progression from ordinary variation. GRAPE, designed for glaucoma follow-up, contains linked visual fields, fundus images, OCT measurements, and clinical data, but only 1115 follow-up records from 263 eyes [57]. Such resources are valuable and still modest compared to cross-sectional image banks.

This scarcity makes leakage especially damaging. Images from the same eye, and usually both eyes of the same person, should not be divided across training and test sets. Information recorded after the prediction time—including updated labels, future visit counts, or treatment escalation—should not enter preprocessing or model development. Otherwise, apparent temporal accuracy partly reflects knowledge of the future.

Longitudinal clinical analysis also requires a prespecified inferential unit. When both eyes are included, analyses should account for within-person dependence, report whether trajectories are eye-specific or patient-level, and prespecify how discordant change between fellow eyes will be interpreted. This is important because unilateral local disease, asymmetric treatment or image-quality differences could otherwise be mistaken for systemic change.

### 6.3. Multimodal Series Are Informative Because They Disagree

Structural OCT, OCTA, tear measurements, home imaging, and systemic variables operate on different clocks. Artificial intelligence can combine these streams, but it cannot decide by itself what their disagreement means. A longitudinal glaucoma model that fused fundus photographs with clinical narratives showed that image and text sequences can estimate present and future visual fields [58]. Its target, however, was a defined local functional outcome. Systemic monitoring is less constrained.

Blood pressure, glycaemia, medication, exercise, sleep, and inflammation may all change between ocular visits. Linking a retinal measurement to the nearest laboratory value may be convenient but biologically wrong if the relevant exposure occurred earlier. Home OCT increases temporal resolution on the ocular side [45] without guaranteeing equally dense systemic data. Missingness also has structure: patients scan less because of illness, poor vision, device difficulty, or loss of motivation, while clinic visits become more frequent when deterioration is suspected.

Multimodal modelling is therefore most persuasive when it tests a specific temporal proposition—for example, whether reduced vascular reactivity precedes neural thinning. Feeding every available variable into one network may improve discrimination while weakening the biological argument.

### 6.4. Personalization, Treatment, and Change Points

A population model provides the expected course; the patient’s previous measurements determine whether the current observation is unusual for that person. This division is more realistic than a universal threshold, but it requires the model to accommodate different starting levels, ageing rates, and measurement variances. One poor scan should not redefine the baseline.

Change-point detection may be more useful than a single average slope. An abrupt departure can follow an acute systemic event, treatment change, inflammatory episode, or local ocular complication. The algorithm may detect the departure, but attribution still requires contemporaneous clinical information.

Treatment complicates observational learning. Once management changes in response to risk, the future course is no longer the untreated course. High-risk patients may improve because they received more intensive care, leading a naive model to learn that severe baseline disease is protective. Intervention-responsive measures such as retinal calibre and flicker responses [50,51,52] should therefore be analysed with treatment timing and sustained exposure made explicit.

### 6.5. Interpretability, Versioning, and Prospective Use

Longitudinal interpretability asks why the estimate changed between visits. A heat map on one image is not sufficient. Clinicians need to know whether the new output was driven by vessel geometry, layer thinning, image quality, or newly available clinical data. Attention maps can locate regions associated with prediction [54], but they do not establish causation.

The model itself may change. Retraining, new preprocessing, a software update, or a different device can alter the score assigned to an unchanged patient. Version control and bridging analyses are therefore part of clinical measurement, not merely software administration. Calibration should also be checked over time; a model can preserve ranking while systematically exaggerating the speed or probability of progression. After a material change in camera, OCT device, segmentation software, preprocessing pipeline or model version, previously established personal trajectories should not be assumed to remain directly comparable; an overlap period or formal bridging study should be required before longitudinal interpretation continues.

Foundation models add a further layer to this problem. RETFound showed that self-supervised pretraining on large retinal image datasets can improve generalizable disease detection and prognosis across several ocular tasks [59]. Such models are promising for future longitudinal oculomics because they may reduce dependence on small labelled datasets. They also make versioning, calibration and external validation more important: a model that has learned broadly useful retinal representations still has to prove that its output changes reproducibly when the patient changes, and remains stable when the patient does not.

Evidence from ophthalmology clinical trials of artificial intelligence also reinforces the need for prospective evaluation, transparent endpoints and clinically relevant deployment pathways, rather than relying only on retrospective image-level performance [60].

The decisive test is prospective. A locked model should generate predictions before outcomes are known and be judged by consequences: earlier recognition of important change, fewer unnecessary visits, better allocation of treatment, or no improvement at all. Dynamic oculomics needs AI because the data are irregular and heterogeneous. It does not need complexity for its own sake.

## 7. Validation and Diagnostic Qualification: From Measurable Change to a Clinical Decision

A statistically detectable ocular change is not yet a clinically useful one. This is a common source of confusion in biomarker research. A difference may be real but too small to matter. It may be associated with an outcome but unable to improve prediction. It may predict risk but fail to suggest any useful action. Therefore, the path from measurable change to clinical decision must be made explicit.

Dynamic oculomics requires several levels of qualification. First, the instrument and the analytical pipeline must measure consistently. Second, the observed trajectory must correspond to a plausible biological process. Third, this interpretation must remain valid in populations and settings different from those in which it was first described. Finally, the information must improve a clinical or research decision. Failure at any one of these levels limits what can be claimed.

### 7.1. Reliability, Agreement, and Meaningful Change

Intraclass correlation coefficients are useful for reliability studies, but a high coefficient can coexist with within-person error large enough to hide a small biological signal. The form of the coefficient, use of single or averaged measurements, and whether consistency or absolute agreement is assessed should be reported [61].

Agreement is the more direct question when two visits or devices are expected to yield interchangeable numbers. Bland–Altman agreement analysis is used to examine systematic bias and the expected range of differences, but it must be reported with sufficient methodological detail to be interpretable [62]. Correlation alone can remain high even when one system consistently reads higher than another.

Each modality therefore needs a minimal detectable change expressed in clinically interpretable units and estimated for the relevant device, anatomical region, and pipeline. Several baseline acquisitions may be justified when the expected signal is small, although averaging helps only if the tissue is stable during that period. Short-term repeatability also cannot guarantee continuity over years of hardware, software, and operator change.

### 7.2. Design and Modelling Must Match the Process

A repeatability study needs closely spaced measurements under stable conditions. A challenge experiment needs dense sampling around the stimulus. Progression requires follow-up long enough to separate drift from short-term variation, while a treatment study should sample when the proposed response could plausibly occur. Using visits simply because they are available can leave the design mismatched to the biology.

Routine data add an informative visiting process. Patients who worsen or receive treatment are seen more often, and mixed-effects estimates can be biassed when visit timing depends on health status [63]. Planned and unplanned visits, missed appointments, treatment changes, and home-monitoring adherence belong in the analysis rather than in an administrative appendix.

Two observations show a difference, not a trajectory. Mixed-effects models accommodate repeated measurements and unequal follow-up, but a precise linear slope can still conceal stability followed by sudden decline. Splines and change-point models offer flexibility at the cost of overfitting, especially when each participant contributes few observations.

When a changing ocular marker is related to a later event, entering an estimated slope into a survival model treats the slope as error-free. Joint models can link the evolving biomarker and event process more directly [64]. The chosen association—current value, cumulative burden, slope, or acceleration—should follow a biological hypothesis rather than whichever specification reaches significance.

### 7.3. External Validation and Added Value

External validation should reproduce the longitudinal task, not merely use images from another institution. The number and spacing of visits, prediction horizon, outcome definition, treatment setting, and acquisition process should be comparable. A model developed in a tertiary clinic may not transport to population screening, where prevalence is lower and repeat imaging less regular.

Calibration matters as much as ranking. A model may order patients correctly while overstating absolute risk or the speed of deterioration, which becomes consequential when the output changes follow-up intervals. Temporal validation is also needed because cameras, treatment standards, and referral pathways evolve. Recalibration creates a new model version and requires an explicit bridge to earlier outputs.

Independent association is not the same as useful improvement. The relevant comparator includes the information clinicians already possess: blood pressure, glycated haemoglobin, renal function, neurological findings, or organ-specific imaging. Decision-curve analysis can test whether an ocular trajectory yields net benefit across plausible action thresholds [65], but the threshold itself depends on the harm of missed disease, the burden of further testing, and the availability of effective intervention.

### 7.4. Biomarker Role and Clinical Action

Terminology should reflect evidence. The BEST framework distinguishes prognostic, predictive, monitoring, response, safety, and other biomarker roles [66]. A variable may track disease without changing after treatment; a response marker may change without predicting benefit; and a predictive marker may identify likely responders without being useful for serial monitoring.

For devices and software that collect repeated biometric measurements, the V3 framework offers a complementary language. It separates verification of the technology, analytical validation of the derived measure, and clinical validation in a defined context of use [67]. This distinction is directly relevant to home imaging, contact lens IOP sensors and algorithm-derived ocular trajectories.

Surrogate-endpoint status is harder still. The treatment-induced change in the ocular measure should reliably predict the treatment-induced change in a clinically meaningful outcome [21]. Prognostic association or response in one trial is insufficient.

Clinical action has to be defined before a threshold is promoted. An alert may trigger repeat ocular imaging, laboratory testing, referral, treatment modification, or reassurance. Each pathway carries different costs and risks. A sensitive system that produces frequent low-value referrals can be accurate and still be harmful.

### 7.5. Prospective Impact and Transparent Reporting

Retrospective accuracy cannot show whether displaying the trajectory improves care. A prospective impact study should identify who receives the result, how uncertainty is shown, what action is recommended, and what happens when the clinician disagrees. Outcomes should include time to appropriate treatment, unnecessary referrals, workload, cost, anxiety, and unequal access.

A silent prospective phase is often useful: the model runs in real time without influencing care, allowing calibration, missingness, and technical failure to be examined before clinicians see the output. Live evaluation can then test the performance of the human-AI team rather than the algorithm in isolation.

TRIPOD + AI provides current reporting guidance for prediction models, while PROBAST + AI addresses quality, risk of bias, and applicability [68,69]. For longitudinal ocular models, reports should also state how visits were selected, whether both eyes were included, how within-person dependence and device changes were handled, and whether future information entered preprocessing. Few ocular trajectories have completed this full pathway. That is a limitation of the evidence, not a reason to relax the standard.

## 8. Ethics, Governance, and Responsibility: What Follows an Ocular Alert?

Dynamic oculomics changes the ethical problem created by ocular biomarkers. A one-time prediction may generate an incidental risk estimate. Repeated monitoring creates a continuing relationship: data are collected, trajectories are recalculated, and an alert may appear long after the original eye examination. At that point somebody must decide whether the alert is credible, whether the patient should be informed, and what should happen next. These questions fall within the broader ethical requirements of autonomy, transparency, accountability, inclusiveness, safety, and sustainability for artificial intelligence in health [70].

### 8.1. Consent, Disclosure, and Incidental Findings

An image obtained for diabetic retinopathy screening or glaucoma follow-up may later be analysed for cardiovascular or cognitive risk. Patients may accept secondary research use without expecting those inferences to be returned. Consent should therefore distinguish immediate care, storage and research, and generation of additional systemic risk estimates. European data-protection law treats health data as a special category and requires an appropriate legal basis, safeguards, and purpose specification [71].

The option not to receive uncertain secondary inferences deserves consideration when the result lies outside the original purpose of care and no validated pathway of benefit exists [70]. Communication should avoid converting a probabilistic trajectory into a diagnosis. The uncertainty, population of validation, plausible ocular explanations, and next reasonable step all belong in the discussion.

A return policy should exist before incidental findings arise. Confirmed findings with clear clinical significance differ from signals that need repeat acquisition, specialist review, or no individual disclosure. Because an apparent trajectory can reflect image quality, device change, or local ocular disease, confirmation will often begin with a standardised ocular examination rather than immediate systemic referral.

### 8.2. Privacy, Equity, and Secondary Data Use

Longitudinal image series linked to molecular samples, treatment records, wearables, and outcomes reveal more than a single photograph. Pseudonymisation reduces risk but does not make a richly dated clinical history anonymous. Access controls, audit trails, data minimisation, and defined retention periods remain necessary [71,72]. Derived scores require governance too: it should be clear whether they enter the medical record, can be reused for training, and can be accessed or contested by the patient.

The European Health Data Space provides a framework for primary and secondary use of electronic health data, including secure processing environments and oversight of access [72]. Easier access can support larger longitudinal cohorts, but it does not justify every new inference.

Repeated imaging is not equally available. Specialist centres, home-monitoring technology, connectivity, physical ability, and sustained engagement shape who contributes data and who benefits. Calibration, false-alert rates, missed progression, and image rejection should be examined across clinically relevant groups. An accurate alert offers little benefit when confirmatory care is inaccessible.

### 8.3. Regulation, Responsibility, and Post-Deployment Monitoring

Regulatory status depends on intended use. Exploratory research software differs from a system that alters surveillance or triggers referral. In Europe, the Artificial Intelligence Act and Medical Device Regulation may both be relevant when an AI system performs a medical function [73,74]. Intended users, target population, action threshold, and workflow position must be explicit.

Longitudinal tools also face model updates. Retraining or revised segmentation can change a patient’s apparent course without biological change. Good machine-learning-practice principles therefore emphasise representative data, independent testing, evaluation of the human-AI team, version control, and post-deployment monitoring [75].

Responsibility cannot be assigned to ‘the system’. Ophthalmologists understand image quality and local disease; general practitioners and specialists place the alert in systemic context; developers control the model. A service needs named responsibility for review, communication, escalation, and closure. DECIDE-AI provides guidance for early live evaluation of clinical decision-support systems, including workflow and human-factor effects [76]. Patients should know who is overseeing the programme and whom to contact when an alert is issued.

## 9. Practical Guidelines for Diagnostic Use of Personalised Ocular Trend Analysis

The following points translate the preceding arguments into practical rules for diagnostic study design and clinical use. They are intentionally restrictive: dynamic oculomics should begin with the question to be answered, not with the number of variables that can be extracted.

1. Define the temporal diagnostic question. Specify whether the aim is classification, monitoring, treatment-response assessment, early warning, or trial enrichment. The expected change, time horizon and possible decision must be explicit.
2. Select the modality according to timescale. Structural OCT is suited to slow neuroaxonal loss, OCTA to microvascular change, challenge tests to reserve and recovery, tear biomarkers to faster surface molecular signals, aqueous humour to selected intraocular molecular profiling when sampling is clinically justified, continuous IOP monitoring to circadian pressure-related patterns, and home devices to dense sampling.
3. Establish both population reference and personal baseline. Population ranges describe how unusual the starting value is. The personal baseline describes whether that value is moving. More than one technically adequate baseline measurement is preferable when the expected change is small.
4. Quantify measurement error and physiological variability. Reliability coefficients are useful, but clinicians need minimal detectable change in the same units used for interpretation, such as micrometres, vessel density, IOP-related output or biomarker concentration [61,62].
5. Standardise acquisition and record device/software details. Time of day, image quality, signal strength, scan placement, pupil status, medication timing, device type, software version and local ocular events should be recorded because they may alter the apparent trajectory [7,8,9,10,33,34,35,36,37,38,42,43].
6. Record systemic exposures and interventions with timing. Ocular change can be interpreted only in relation to the systemic process it is meant to represent. Blood pressure, glycaemic control, medications, exercise, weight loss, inflammatory activity and neurological events should be documented with adequate temporal resolution [46,47,48,49,50,51,52].
7. Model the trajectory explicitly. The analysis should distinguish level, slope, variability, acceleration, change point, response and recovery. Two measurements show a difference; they do not define a reliable trajectory.
8. Confirm abnormal change before interpretation. A suspected change should be compared with expected variability and minimal detectable change. Uncertain signals should be repeated or corroborated by a complementary modality before they are used diagnostically.
9. Compare ocular trajectories with conventional systemic markers. Concordance may support interpretation, but discordance can be more informative when confirmed. The ocular course should be examined alongside laboratory tests, clinical examination, systemic imaging and treatment history.
10. Act only when the pathway is validated for the intended decision. The measurement or model should be tested under the intended observation schedule, compared with simpler alternatives, externally calibrated, and evaluated for clinical or research benefit [63,64,65,66,67,68,69]. An alert also requires a responsible clinician and a defined pathway for confirmation, referral, communication and closure [70,71,72,73,74,75,76].

Economic sustainability is part of this validation pathway. Dynamic oculomics may require repeated imaging, longer acquisition time, device maintenance, software licences, data storage, clinical review, confirmatory testing and management of false alerts. These costs should be considered together with potential benefits, such as better targeting of follow-up, avoidance of unnecessary visits and earlier recognition of relevant progression. Health-economic reporting frameworks such as CHEERS 2022 and recent analyses of artificial-intelligence screening for diabetic retinopathy show that diagnostic accuracy alone does not determine real-world value; cost-effectiveness depends on setting, prevalence, willingness-to-pay thresholds, referral burden and the balance between sensitivity and specificity [77,78].

**Figure 3:**
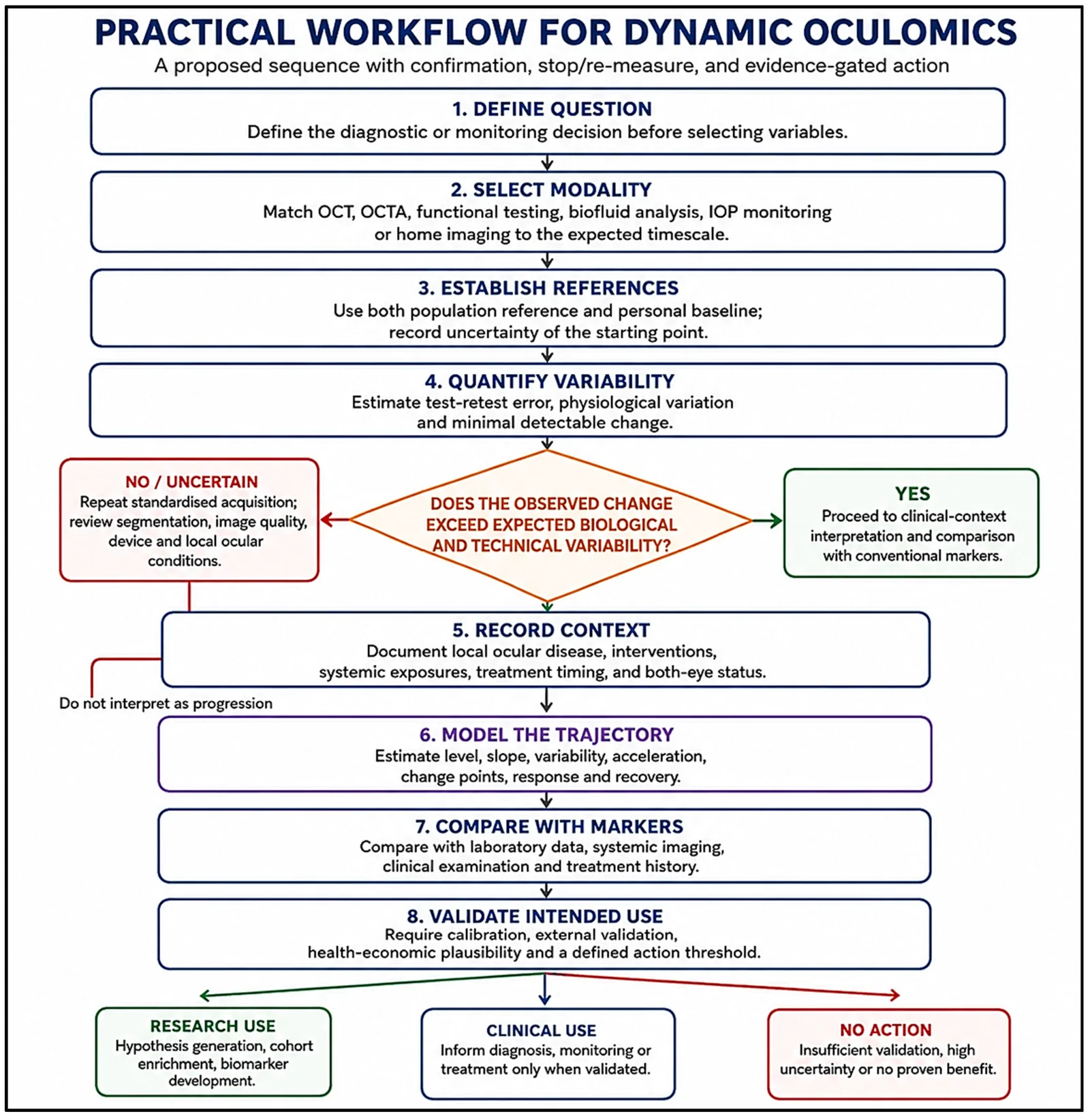
Workflow for diagnostic use of dynamic oculomics. The revised workflow moves from the diagnostic question to modality selection, baseline definition and variability assessment, then introduces a decision node asking whether the observed change exceeds expected biological and technical variability. No or uncertain findings lead to repeat standardised acquisition and review of segmentation and image quality; confirmed findings proceed to context recording, trajectory modelling, comparison with conventional markers and evidence-gated action.

The following workflow (Figure 3) integrates the principal technical, biological, analytical, economic and governance requirements discussed above into a proposed sequence for evaluating dynamic ocular markers.

## 10. Conclusions

Oculomics, in its traditional use, has established that ocular images, ocular biofluids and functional ocular tests may contain measurable information about systemic health. This approach is mainly cross-sectional. It compares one value with a population or converts it into a diagnostic or prognostic score. Such information can be useful for screening, risk enrichment and baseline classification, but it cannot determine whether the biological state of the same patient is changing.

Oculomic kinetics, or dynamic oculomics, begins from the opposite side of the diagnostic problem. It asks how the same ocular variable behaves within the same person over time. The relevant information is not only the value itself, but also the baseline, the variability around that baseline, the rate of change, the response to perturbation, the recovery phase and the modification produced by treatment.

The two approaches should not be placed in competition. Static oculomics is most useful when the aim is classification, screening or broad risk estimation. Dynamic oculomics is more appropriate when the aim is monitoring, treatment-response assessment, diagnostic reassessment, or identification of discordance between conventional markers and tissue-level behaviour. Both approaches are useful, but they answer different questions.

The field does not need to prove that the eye mirrors the whole body. It needs to identify those settings in which the personal ocular course carries diagnostic information that cannot be obtained as easily, as early, or as safely elsewhere. In this more limited but clinically realistic sense, dynamic oculomics may become part of diagnostic medicine: not by replacing conventional biomarkers, but by adding a repeatable tissue-level history to the individual patient profile.

## Figures and Tables

**Table 2:** Provisional functional roles of a dynamic ocular marker.

Role	Meaning	Typical Timescale	Possible Ocular Example	Main Interpretive Warning
State marker	Reflects current physiological conditions.	Minutes to days	Vessel calibre, choroidal thickness, tear mediator level	May fluctuate without indicating damage.
Strain marker	Suggests recruitment or loss of compensatory reserve.	Minutes to months	Reduced flicker response, abnormal recovery after challenge	Reserve failure may precede structural change but is not organ-specific.
Damage marker	Records persistent structural or functional loss.	Months to years	RNFL or GCIPL thinning, capillary loss, persistent lesion	May reflect past exposure rather than current disease activity.
Response marker	Changes after treatment or defined intervention.	Days to months	Improved vascular reactivity, stabilisation of thinning, molecular change in tears	Treatment response does not establish surrogate-endpoint status.

**Table 3:** Ocular modalities for dynamic oculomics according to temporal question and current evidence maturity.

Modality	Temporal Use	Main Variables	Main Limits	Current Use	Current Evidence Level
Fundus photography	Slow vascular geometry and lesion change	Calibre, tortuosity, fractal dimension, lesions	Magnification, centration, camera changes, image quality	Opportunistic vascular-geometry follow-up	Limited longitudinal systemic use; stronger in imaging cohorts
Structural OCT	Slow tissue loss or stabilisation	RNFL, GCIPL, retinal and choroidal thickness	Segmentation, registration, device/software changes	Neuroaxonal and retinal disease monitoring	Established in local ocular disease; selected neurological applications
OCT angiography	Microvascular drift or short-term vascular change	Vessel density, FAZ, flow voids, plexus metrics	Signal strength, motion, segmentation, projection artefacts	Selected microvascular studies	Supported by repeatability studies and selected cohorts
Functional challenge tests	Reserve, response and recovery	Flicker dilation, pupillary response, electrophysiology	Stimulus standardisation, systemic state, repeatability	Neurovascular coupling and vascular-reserve studies	Limited longitudinal human evidence
Tear analysis	Molecular change over hours to weeks	Proteins, cytokines, soluble mediators	Collection method, dilution, storage, analyte stability	Exploratory inflammatory and treatment-response studies	Exploratory for longitudinal systemic monitoring
Aqueous humour analysis	Selected intraocular molecular profiling	Proteins, cytokines, metabolites, trace elements, transcripts	Invasive sampling, surgical context, low volume, barrier status	Selected intraocular disease and treatment studies	Emerging; mostly cross-sectional or opportunistic longitudinal use
Home/portable imaging	Dense sampling and response curves	Repeated OCT or image-derived outputs	Adherence, self-acquisition, artefacts, missingness	Selected retinal diseases	Established in selected retinal disease; systemic use exploratory
Continuous IOP monitoring	Circadian pressure-related pattern	24 h contact-lens sensor output, acrophase, amplitude, nocturnal profile	Arbitrary units, ocular-surface tolerance, device specificity	Glaucoma risk stratification and monitoring	Established in selected glaucoma studies; not systemic

## Data Availability

No new data were created or analysed in this study. Data sharing is not applicable to this article.

## Abbreviations

AI, artificial intelligence; AMD, age-related macular degeneration; BEST, Biomarkers, EndpointS, and other Tools; DRCR, Diabetic Retinopathy Clinical Research Network; FAZ, foveal avascular zone; GCIPL, ganglion cell–inner plexiform layer; OCT, optical coherence tomography; OCTA, optical coherence tomography angiography; RNFL, retinal nerve fibre layer.
